# A Pilot Study of Two Different Constraint-Induced Movement Therapy Interventions in Children With Hemiplegic Cerebral Palsy After Botulinum Toxin Injection During Preschool Education

**DOI:** 10.3389/fped.2020.00557

**Published:** 2020-10-22

**Authors:** Chin-Lung Wu, Su-Fen Liao, Chi-Hsin Liu, Yu-Ting Hsieh, Yi-Ru Lin

**Affiliations:** ^1^Division of Occupational Therapy, Department of Physical Medicine and Rehabilitation, Changhua Christian Hospital, Changhua City, Taiwan; ^2^Division of Pediatric Rehabilitation, Changhua Christian Children's Hospital, Changhua City, Taiwan; ^3^Department of Physical Medicine and Rehabilitation, Changhua Christian Hospital, Changhua City, Taiwan; ^4^Department of Physical Therapy, HungKuang University, Taichung City, Taiwan; ^5^Division of Physical Medicine and Rehabilitation, Lukang Christian Hospital, Changhua City, Taiwan

**Keywords:** cerebral palsy—diagnosis, therapy, constraint-induced movement therapy (CIMT), botulinum toxin (BoNT), rehabilitation—ot/pt, preschool education

## Abstract

**Introduction:** To establish a pilot study on applying two low dose (40 h) constraint-induced movement therapy (CIMT) interventions in children with hemiplegic cerebral palsy (CP) after botulinum toxin (BoNT-A) injection during preschool education.

**Methods:** Five children with spastic CP (mean age: 5.31 years; Gross Motor Function Classification System level I and II) undergoing regular BoNT-A injections and rehabilitation programs were included. Participants were randomly allocated to one of two CIMT programs (40 h): a 2-week 4-hours/day CIMT program and a 4-week 2-hours/day CIMT program. One CIMT program was performed 1 month after a BoNT-A injection, and then the second program was implemented with the next injection. The outcomes were measured by changes in Goal Attainment Scaling (GAS), the grasp and Visual-Motor Integration (VMI) test in Peabody-Developmental Motor Scales (PDMS), the self-care scale on the Functional Skill Scale, and the Caregiver Assistance in Chinese Version of Pediatric Evaluation of Disability Inventory (PEDI-C), Anxiety and Oppositional Defiance Problems of Achenbach System of Empirically-Based Assessment before and after the CIMT interventions, and at every 2 months' follow-up thereafter.

**Results:** The mean age of the participants was 5.31 years, BMI was 16.7 (kg/m^2^), VIQ was 86.4 ± 8.5, and dose of BoNT-A injection in the upper limb was 42 ± 26.6 units. Grasp, VMI, and self-care on the Functional Skill Scale were significantly better in the 4-week 2-hours/day CIMT program (*p* < 0.001, *p* = 0.001, *p* < 0.001). GAS, grasp, VMI, two 2 self-care scales of PEDI were significantly improved after the CIMT programs, and improvement continued for up to 4 months after the programs. There was no clinical evidence showing changes in the scores for anxiety and oppositional defiance problems during the study period.

**Conclusions:** The preliminary findings, although limited, suggest a potential therapeutic role for the school-based CIMT program after BoNT-A injection. The 4-week 2-hours/day CIMT program might be better than a 2-week 4-hours/day program in terms of self-care and hand function when performed in kindergarten in this pilot study. Furthermore, this pilot study provides valuable information; therefore, it is crucial to include more CP children and blinded assessors for hand function and ADL in the future study.

## Introduction

Cerebral palsy (CP) is the most common physical disability in childhood, and 39% of children with CP have hemiplegia (1–3). Hemiplegic CP children develop 90% of their hemiplegic hand function between the ages of 3 and 7 years (4), so preschool intervention is important to facilitate further development of hand function (5). Case-Smith, concluded that visual-motor interventions for children with developmental delays could improve visual-motor performance in the short term, and occupational therapy embedding both behavioral and learning principles appears to have positive effects on preschool children (3–5 years old) (6). Botulinum toxin A (BoNT-A) has been widely used to relieve the spasticity of CP children (7, 8). Occupational therapy after BoNT-A injection can improve hand function and functional skill performance in hemiplegic CP children (3). Constraint-induced movement therapy (CIMT) can facilitate hemiplegic hand function in stroke patients and CP children (9, 10). CIMT is child-active repetitive and structural training in the use of the hemiplegic upper limb by constraining the dominant hand (11). DeLuca et al., reported that pediatric CIMT at both moderate (63 h) and high doses (126 h) produced positive effects, and their findings refuted the hypothesis of differential dosage benefits (12). Novak, concluded that a period of 30–60 h of therapy within a 6–8-week period is needed to be effective (3).

Child-active approaches, for example, goal-directed training in hand function tasks (e.g., typing), that are designed to meet a goal meaningful to the child (3), are consistent with neuroscientific evidence with regard to inducing maximal neuroplasticity (13). Goal Attainment Scaling (GAS) is a method of scoring the extent to which the patient's individual goals are achieved in the course of intervention. GAS was first introduced in the 1960s by Kirusek and Sherman (14) for assessing outcomes in mental health settings (15, 16). GAS provides a useful measure of functional gains in response to rehabilitation and is more sensitive than global measures such as the Barthel Index (15). GAS could also offer the opportunity for a single interval measure with which to assess response to intervention with BoNT-A (15, 17). GAS has also shown promising qualities in pediatric rehabilitation (18).

This study was designed to be a pilot study employed for 4-week 2-hours/day and 2-week 4-hours/day CIMT (40 h) interventions in children with hemiplegic CP after a BoNT-A injection during preschool education, and to compare the effects and psychological stress of CP children relative to the two CIMT programs. The main hypothesis was that a low-dosage school-based CIMT (40 h) program could be performed smoothly in preschool and produce benefits in hand function and activities of daily living (ADL). The second hypothesis was that 2-week 4-hours/day CIMT would produce significantly greater benefits than 4-week 2-hours/day CIMT and less psychological stress.

## Materials and Methods

This study used a randomized crossover design with a “wash-out” period, and was conducted from June 2014 to December 2016. The study was approved, and ethical clearance was obtained from the hospital Institutional Review Board (IRB 130210). All participants and their parents provided written informed consent for participation.

Children with hemiplegic or quadriplegic spastic CP who were in a regular rehabilitation program with BoNT-A injections were invited to participate in this study. The enrolled children were classified as Gross Motor Function Classification System level I or II, were aged between 3 and 6 years old, with at least below average mentality, and were in mainstream preschool education; the extension of their hemiplegic wrist and metaphalangeal joint was >10°. Children were excluded if they: (a) could not understand or cooperate with the CIMT program, (b) had joint contracture of an upper limb, and (c) could not actively extend their hemiplegic wrist. Based on a repeated ANOVA comparison of changes using an effect size of −0.8 for both groups, a significance (alpha) level of 0.05, and 80% power, we required six children for this pilot study.

Participants were randomized using a computerized random number and crossover-allocated to two different CIMT programs: one was a 2-week 4-hours/day program, and the other a 4-week 2-hours/day constraint program in preschool education.

After at least a 7-month “washout period,” the children were crossed over to another CIMT program (19). The CIMT program was performed 1 month after BoNT-A injection at the involved upper limb, and was changed to the other program with the next BoNT-A injection. The CIMT program was carried out by preschool teachers during regular school activities, and an arm sling worn on the non-involved hand was used as a restraint. The child's occupational therapist was responsible for treatment planning and adjusting the plan according to the school programs. All children were asked to maintain their ordinary treatments during the study period. Intramuscular injections of BoNT-A were performed by the same physiatrist (Dr. SF Liao), using 0.5–4 units of BoNT-A/kg/muscle group (Allergan PLC, Dublin, Ireland); the injection intervals depended on the spasticity condition of the children.

The outcomes were measured by any change in two playing goals and two self-care goals of the GAS, grasp, and visual-motor integration (VMI) in the Peabody-Developmental Motor Scales II (PDMS II), 73 functional skills and eight caregiver assistance self-care scales in the Chinese Version of the Pediatric Evaluation of Disability Inventory (PEDI-C), and anxiety and oppositional defiance problems in the Caregiver-Teacher Report Form (C-TRF)-Diagnostic and Statistical Manual (DSM)-oriented scales of the Achenbach System of Empirically-Based Assessment (ASEBA). Measurements were taken just before the BoNT-A injection (T0), 1 month after the injection (T1), shortly after the CIMT intervention (T2), and at 2 months (T3) and 4 months (T4) after CIMT. The measurements of PDMS II and GAS were performed by the child's occupational therapists, the PEDI-C records were maintained by the child's caregivers, and the C-TRF measurements were performed by the child's caregivers and school teachers. The measurements could not be blinded because the assessors (teachers, caregivers, and therapists) had to know the programs and how to operate them.

GAS included two playing goals and two self-care goals. The goals were decided by the occupational therapist, the preschool teachers, and the main caregiver. The goals were weighted by applying the factor of importance × difficulty, where importance of the goal to the patient was graded as 1 = fairly important, 2 = very important, and 3 = extremely important. Difficulty of achieving the goal was rated as 1 = probable, 2 = possible, and 3 = doubtful. Baseline scores were allocated as −1, if the goal was achieved as predicted, then scored 0 when evaluated at 2 and 4 months after CIMT. Achievement above the level predicted was scored at +1 (“somewhat better than expected”) or +2 (“much better than expected”). No change or achievement below the expected level was scored as −1, and a worsening of the target function was scored as −2. GAS was calculated for the aggregated score of each patient's goals by applying the formula recommended by Kiresuk and Sherman (14).

The fine motor skills of the affected upper limb were evaluated using the grasping and VMI scores of the PDMS II. These scores can gauge improvement after the CIMT program. The C-TRF (1.5–5) DSM-oriented scales of the ASEBA can assess the children's behavioral/emotional problems from the perspectives of multiple informants (20). The anxiety and oppositional defiant problems in the C-TRF were used to evaluate the emotional stress of the CP children during the study period.

The Wechsler Preschool and Primary Scale of Intelligence-Revision-Chinese-language version (21) is used to assess the intelligence of children aged between 3 and 7 years; the scale includes a full-scale intelligence quotient, verbal intelligence quotient (VIQ), and performance intelligence quotient. The VIQ is used to determine intelligence status without interference by motor impairment. Children with a VIQ ≧ 70 were included.

## Statistical Analysis

Descriptive statistics (including mean, range, frequency, and percent) were used for demographic and clinical/treatment factors of interest. Comparisons of the two CIMT programs and the different times were carried out using Generalized Estimating Equations. Differences in the C-TRF-DSM scores between the caregivers and teachers were analyzed by Paired Samples *T*-test.

All analyses were performed using SPSS software version 22.0 for Windows (SPSS Inc., Chicago, IL). A *p* < 0.05 was considered statistically significant.

## Results

Five children finished the study; one child did not finish the program because his family moved to another city. The mean age of the participants was 5.31 years, BMI was 16.7 (kg/m^2^) (22), VIQ was 86.4 ± 8.5, and BoNT-A dose injected into the upper limb was 42 ± 26.6 units. Four children had spastic hemiplegic CP and one child had spastic quadriplegic CP (Table 1). The brain MRI revealed one child had unilateral left periventricular leukomalacia (PVL), three had a porencephalic cyst in the right hemisphere, and one had a porencephalic cyst in the left frontoparietal lobe with PVL.

**Table 1:** Basic characteristics of participants at baseline.

**Parameter**	
No	5
Mean age (y)	5.31 ± 0.84
Female	2
Height (cm)	106.2 ± 4.9
Body weight (Kg)	18.3 ± 2.9
BMI (kg/m^2^)	16.7± 2
Diagnosis, *n*	
Spastic hemiplegia	4
Spastic quadriplegia	1
Etiology-congenital	4
Traumatic	1
VIQ	86.4 ± 8.5
More-affected side, *n*	
Right	2
Left	3
Dose of BoNT-A (units) (number of injections)	
Upper limbs	42 ± 26.6
Pectoralis major	20 (1)
Biceps brachii	30 (1)
Brachialis	21.7 ± 6.8 (6)
Pronator teres	20.6 ± 4.6 (9)
Adductor pollicis	10 (4)
Lower limbs	56 ± 35.7

*BMI, body mass index; VIQ, verbal intelligence quotient; BoNT-A, Botulinum toxin A*.

**Figure 1:**
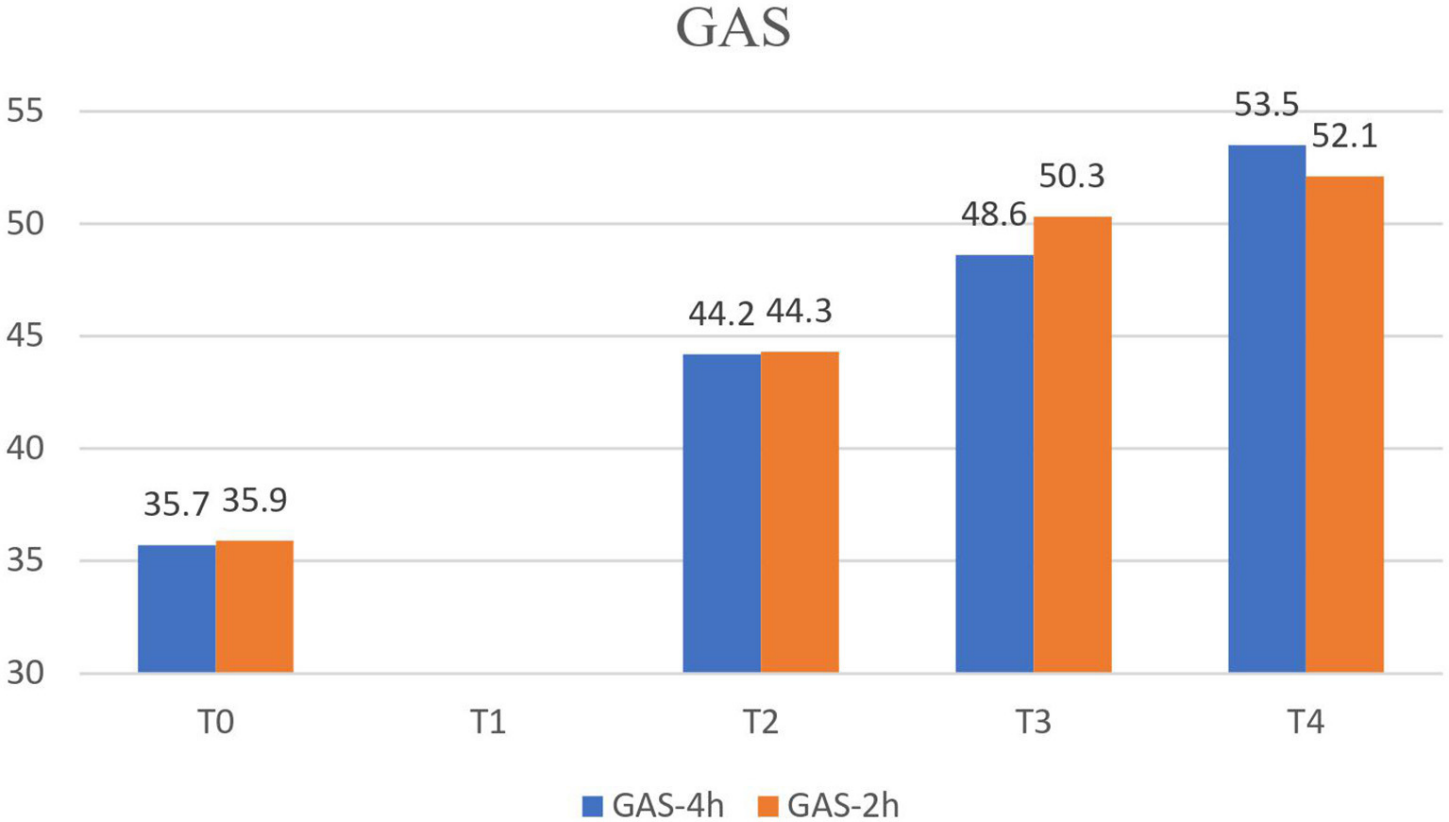
GAS score showed improvement after the CIMT program. GAS, Goal attainment scaling.

The GAS for the two playing goals and two self-care goals was significantly improved after the CIMT programs, and continued to improve until 4 months after CIMT (*p* < 0.001). The GAS especially showed progressive improvement after the CIMT programs (Table 2, Figure 1), but there was no difference (*p* = 0.856) in the GAS between two 2 CIMT programs.

**Table 2:** Summary of outcome measures.

	**T0-Before BoNT-A**		**T1-Before CIMT**		**T2-Shortly after CIMT**		**T3-2-month after CIMT**		**T4-4-month after CIMT**		**GEE**		
	4h^*^2 w	2h^*^4w	4h^*^2 w	2h^*^4w	4h^*^2 w	2h^*^4w	4h^*^2 w	2h^*^4w	4h^*^2 w	2h^*^4w	Program ^*^Time	**Program**	**Time**
PDMS II													
Grasp	44.5 ± 4.9	44 ± 5.6	42.5 ± 4.4^¢^^$^	47.8 ± 2.4	42.5 ± 4.4^‡^^#^	46.2 ± 4.4	46 ± 2^¢^^‡^^§^	46.7 ± 2.1	46.7 ± 2.3^$^^§^^#^	50 ± 2.8	P < 0.001^*^	P = 0.062	P < 0.001^*^
VMI	124 ± 8.5	126 ± 17	109.5 ± 20.6^¢^^$^	130.5 ± 12.5	111.8 ± 21.9^‡^^#^	124.8 ± 19.6	123.7 ± 10.7^¢^^‡^	136.7 ± 7.8	121 ± 15.7^$^^#^	141 ± 2.8	P = 0.001^*^	P = 0.202	P = 0.012^*^
PEDI-C													
Self-care	55 ± 1.4	55 ± 8.5	54.8 ± 5.3^¢^^$^	60 ± 1.2	54.7 ± 6.1^#^	64 ± 3.5	59.7 ± 3.8^¢^^§^	65.5 ± 0.7	61.3 ± 2.3^¢^^§^^#^	66	*P < 0.0*01^*^	P =0.269	P < 0.001^*^
Caregiver	20.5 ± 0.7	20.5 ± 7.8	22 ± 2.7^&^^¢^^$^	24 ± 9.2	24.7 ± 3.1^&^^#^	23.7 ± 7.5	25.3 ± 2.3^¢^	33.5 ± 6.4	28.2 ± 6.7^$^^#^	30	P = 0.153	P = 0.267	P < 0.001^*^
assistant													
GAS	35.7 ± 0.3	35.9 ± 0.4			44.2 ± 7^†^*^‡^^#^*	44.3 ± 5.3^†^*^‡^^#^*	48.6 ± 6.5^‡^^§^	50.3 ± 5.5^‡^^§^	53.5 ± 11.7^§^^#^	52.1 ± 6.8^§^^#^	P = 0.856	P = 0.676	P < 0.001^*^
ABEBA-anxiety													
Teacher			2.67 ± 2.1^&^	3.7 ± 2.9	3.67 ± 2.1^&^	4 ± 2.6^‡^^#^	1.5 ± 0.7	0^‡^^§^	3 ± 2.6	1^#^^§^	P < 0.001^*^	*P* = 0.204	*P < 0.0*01^*^
Caregiver			4.3 ± 1.5	3.3 ± 3.2^¢^^$^	3 ± 2.6^‡^	4 ± 2.6^‡^^#^	4.3 ± 2.5^‡^	2.5 ± 3.5^¢^^‡^^§^	3.5 ± 4.9	5^$^^#^^§^	P < 0.001^*^	P < 0.001^*^	P < 0.001^*^
Oppositional defiant													
Teacher			1.33 ± 1.2	1.7 ± 2.9	2^‡^	1.3 ± 1.1	0^‡^^§^	1	1.7 ± 1.5^§^	1	P < 0.001^*^	P = 0.992	P < 0.001^*^
Caregiver			3 ± 2.6^¢^	1.7 ± 2.9^&^	3.3 ± 2.1	4 ± 2^&^^‡^^#^	2.3 ± 2.3^¢^	3 ± 2.8^‡^^§^	3.5 ± 2.1^#^	4^#^^§^	P < 0.001^*^	P < 0.001^*^	P < 0.001^*^

*BoNT-A, Botulinum toxin A; PDMS II, Peabody-Developmental Motor Scales; VMI, visual-motor integration; PEDI-C, Chinese Version of Pediatric Evaluation of Disability Inventory; GAS, Goal attainment scaling; ABEBA, Achenbach System of Empirically Based Assessment*.

* *P < 0.05*.

† *P < 0.05 the data of T0 compared with T2*.

& *P < 0.05 the data of T1 compared with T2*.

¢ *P < 0.05 the data of T1 compared with T3*.

$ *P < 0.05 the data of T1 compared with T4*.

‡ *P < 0.05 the data of T2 compared with T3*.

# *P < 0.05 the data of T2 compared with T4*.

§ *P < 0.05 the data of T3 compared with T4*.

**Figure 2:**
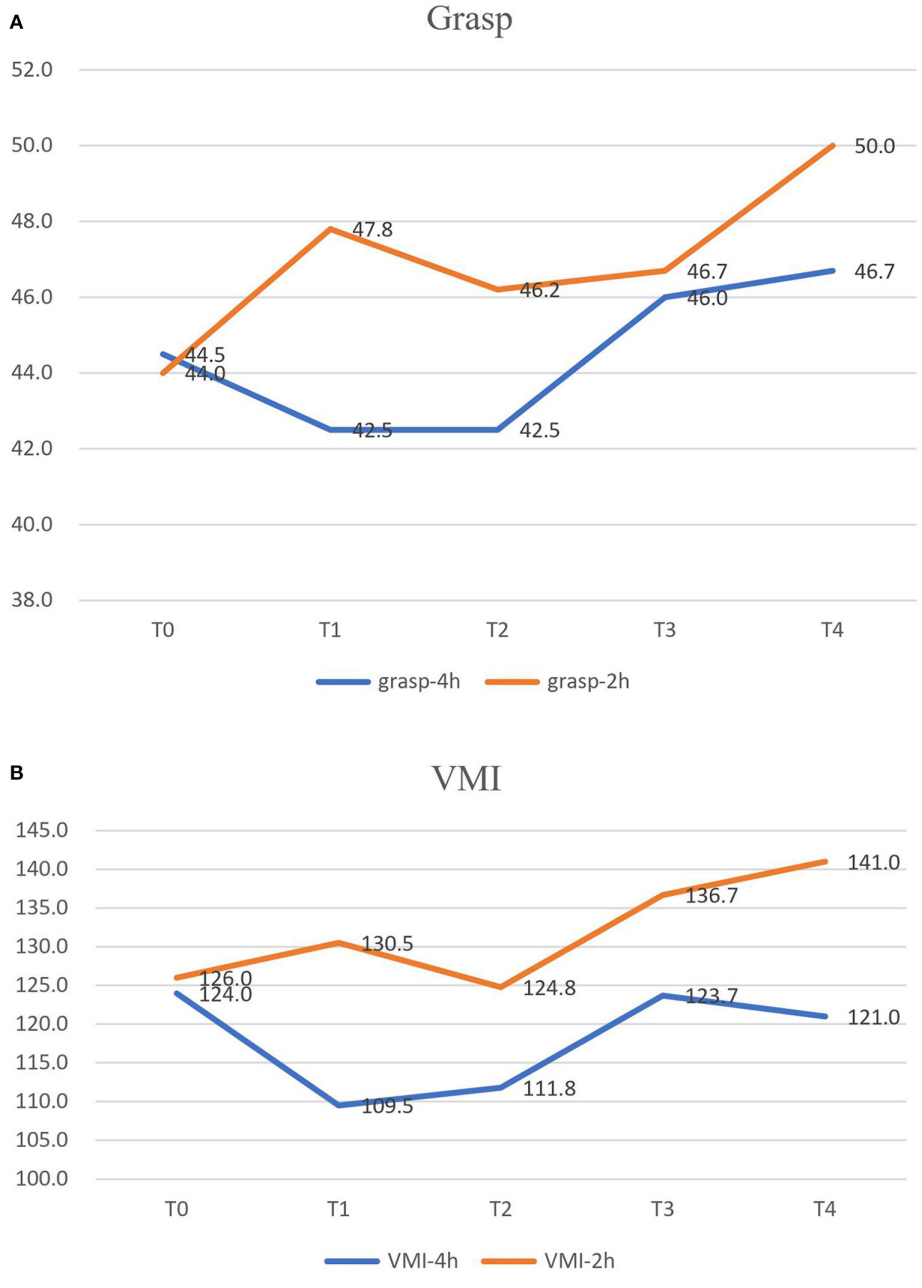
The grasp **(A)** and VMI **(B)** scores of PDMS II were significantly better in the 4-week 2-h CIMT program. VMI, visual-motor integration; PDMS II, Peabody-Developmental Motor Scales II.

The grasp (Figure 2A) and VMI (Figure 2B) scores of the PDMS II were significantly better with the 4-week 2-hours/day CIMT program (*p* < 0.001, *p* = 0.001) (Table 2). The grasp and VMI scores of the two CIMT programs revealed significant improvement after the CIMT program, and continued improving until 4 months after the programs (*p* < 0.001, *p* = 0.012).

**Figure 3:**
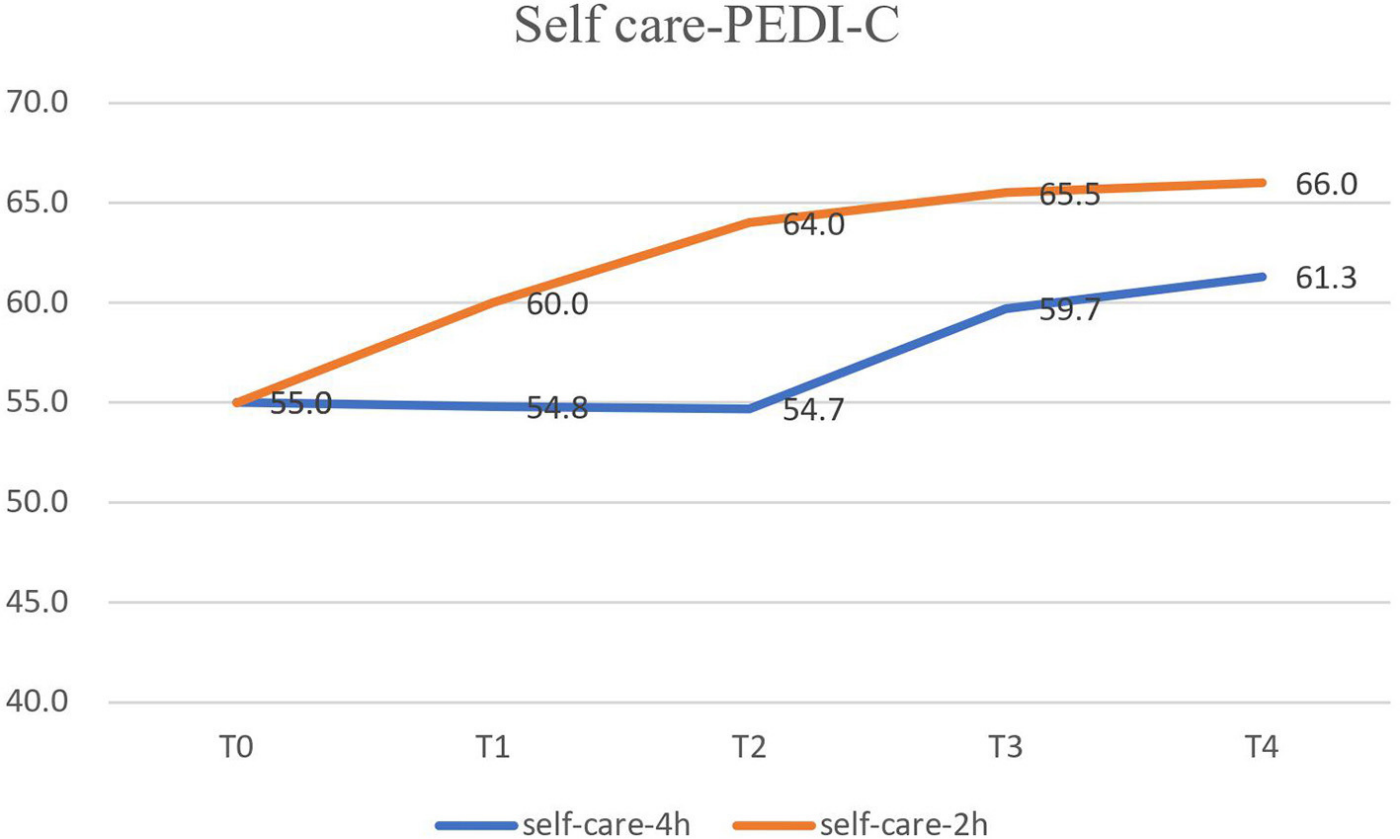
The self-care functional skill of PEDI-C were significantly better in the 4-week 2-h CIMT program. PEDI-C, Chinese Version of Pediatric Evaluation of Disability Inventory.

In terms of ADL, the self-care functional skill score (Figure 3) of the PEDI-C was significantly better with the 4-week 2-hours/day CIMT program (*p* < 0.001, Table 2). However, there was no difference in the caregiver assistance self-care scores between the two CIMT programs (*p* = 0.153, Table 2). The self-care functional skills and caregiver assistance scores were significantly improved at 2 months (T3) and 4 months (T4) after CIMT, but there was no difference in T2 compared with T1 and T0 (Table 2).

The anxiety and oppositional defiant disorder did not achieve clinical significance during the study period. Of interest, the anxiety and oppositional defiant disorder scores reported by the teachers were higher in the 4-week 2-hours/day CIMT (*p* < 0.001, *p* < 0.001) program, but the caregivers reported more stress in the 2-week 4-hours/day CIMT program (*p* < 0.001, *p* < 0.001). The anxiety and oppositional defiant disorder problems reported by the teachers were the highest shortly after CIMT, then gradually improved. In the caregiver's report, anxiety was in the subclinical range during T1 and T3, and oppositional defiant disorder was the highest during T2, then gradually improved (Table 2). We also compared the scores reported by the teachers and caregivers, and found that the caregivers gave higher scores than the teachers (*p* = 0.016, *p* < 0.001).

## Discussion

In our study, we found that the 40-h school-based CIMT program after BoNT-A injection could improve hand function, self-care, and GAS for 4 months, and thus the main hypothesis was corroborated. But the second hypothesis was subverted. The 4-week 2-hours/day CIMT program was more effective than the 2-week 4-hours/day CIMT program in improving hand function and self-care.

Galvin concluded that a combination of BoNT-A and occupational therapy was more effective than occupational therapy alone in reducing impairment and in improving activity-level outcomes and goal achievement. They also concluded that BoNT-A injections should not be used without follow-up therapy intervention (8). In a review article by Novak, CIMT was as effective as Bimanual Training (3). Different researchers have made various modifications to the CIMT program to make it more child-friendly, by reducing both the duration of the intensive training session for the paretic upper extremity and the restraint time for the non-paretic upper extremity. However, Novak suggested that a dose of 30–60 h of therapy within a 6–8-week period is needed to be effective (3). Our study found that 40-h school-based CIMT after BoNT-A is enough to show effectiveness. Most CIMT studies were performed at home (23) or in therapists' rooms (24). We performed the CIMT program in mainstream kindergarten and combined oriented goals with school-activities. Our results showed that both CIMT programs were well-tolerated and were performed smoothly. Another study found that using the 2 hours/day CIMT program in school-based settings can lead to improvements in quality of bimanual skill and movement patterns, but the CIMT program was performed in special education preschool in this study (25).

Goal-directed training that is designed to meet a goal meaningful to the child is an effective intervention in CP rehabilitation (3). In our study, GAS was used for two playing goals and two self-care goals that were chosen by the teacher, caregiver, and therapists. GAS was meaningful to the children and showed the child's progress and improvement after the CIMT program. GAS is a responsive method for individual goal-setting and for treatment evaluation (18), and shows promising qualities for use in CP evaluation.

Grasp and VMI functions were improved after two 40-h CIMT programs, and the improvement lasted 4 months. This is in agreement with a review article by Dong VA (26), in which the CIMT group gained more grasp function in the affected hand than the bimanual training group. Galvin found that a combination of BoNT-A and occupational therapy improved activity level outcomes and goal achievement, but did not improve quality of life or perceived self-competence (8). In our study, school-based CIMT after BoNT-A not only improved goal achievement and hand function, but also improved ADL.

The 4-week 2-hours/day CIMT program was more effective in improving grasp and self-care ADL. It might be that the 2-hours/day CIMT program is more accepted and comfortable for CP children than the traditional CIMT (at least 3 hours/day) program (25). Eliasson reported that the 2-hours/day modified CIMT program improved the children's ability to use their hemiplegic hand, and they experienced little frustration (27). In another study, Eliasson also reported that a 2-hours/day, 2-month Eco-CIMT program carried out by parents and preschool teachers influenced development more than ordinary therapy (19). In contrast, Lin KC et al. reported that a 3.5–4 hours/day, twice a week, 4-week CIMT program yielded higher parent–child dysfunctional interaction immediately after CIMT than that after the control intervention (23). We found that the 4-hours/day CIMT program was more stressful for CP children at home. However, the CP children showed more stress in the 2-hours/day CIMT program at school, which could be because of the longer program (4 weeks).

Overall, there was no significant clinical evidence in the scores for anxiety and oppositional defiant problems. This indicated our treatment programs administered in school did not aggravate the children's stress levels in the long run, as in Lin's study (23). The scores for anxiety and oppositional defiant problems evaluated by caregivers were higher than those in the teachers' report. It might be that the children tend to behave well in school but are more frustrated at home.

The novel finding of our study is that the low-level CIMT program could be performed smoothly by school teachers. Furthermore, the evaluation is multidisciplinary, including not only therapists, but also teachers and caregivers. Most CIMT studies were performed at home (23) or in therapists' rooms (24). Gelkop, N et al. also applied CIMT and hand-arm bimanual intensive therapy (HABIT) in a school-based setting, but the program was performed in a special education preschool and the therapy was handled by occupational therapists.

Our research had several strengths. First, we used a cross-over study design and long wash-out period, which could eliminate the bias of the children's personal and environmental differences, and the residual effect of the last BoNT-A injection. In this way, the children could maintain their regular BoNT-A injection and usual rehabilitation programs, but it would take longer (at least 1 year) to finish the program than in other studies (23, 25, 28). Second, GAS is well-established and has proven to be a useful scale to show the progress and improvement of children in the CIMT program. GAS was chosen by the teachers, caregivers, and therapists; the goals were meaningful to the children and allowed the medical staff and family members to be involved in the program.

This pilot study could be used in larger clinical studies. Cohen's *d* is determined by calculating the mean difference between two CIMT programs to determine the effect size of 0.34. Then, using a repeated ANOVA comparison of change with an effect size of 0.34 for both groups, three measurements, a significance (alpha) level of 0.05, and 80% power, 16 children would be required in a future study.

## Limitations

This study has several limitations. First, the small sample size might limit the power and generalizability of the results to the population of children with CP. Second, we did not use a blinded study design. Therefore, there might be some measurement bias. In a further study with a larger sample size, blinded measurements of PDMS and a measure for spasticity are suggested.

## Conclusions

The results of this pilot study reveal a high rate of completion and adherence in the school-based CIMT program. The preliminary findings, although limited, also suggest a potential therapeutic role for the school-based CIMT program after BoNT-A injection. The 4-week 2-hours/day CIMT program seemed to be better than a 2-week 4-hours/day program in terms of self-care and hand function when performed in kindergarten in this pilot study. However, we must be attentive to the discomfort and emotional stress of the children when using the CIMT program. This pilot study provides valuable information; therefore, it is crucial to include more CP children and a blinded assessor for hand function and ADL in future studies.

## Data Availability Statement

The raw data supporting the conclusions of this article will be made available by the authors, without undue reservation.

## Ethics Statement

The studies involving human participants were reviewed and approved by Institutional Review Board, Changhua Christian Hospital, IRB approval # 130210. Written informed consent to participate in this study was provided by the participants' legal guardian/next of kin.

## Author Contributions

C-LW, S-FL, and C-HL contributed to the study design. C-LW, C-HL, Y-TH, and Y-RL participated in data collection and analysis/interpretation. C-LW and S-FL participated in manuscript preparation. All authors revised and commented on the manuscript. All authors contributed to the article and approved the submitted version.

## Conflict of Interest

The authors declare that the research was conducted in the absence of any commercial or financial relationships that could be construed as a potential conflict of interest.
